# Personal goal facilitation, burnout and work engagement: the role of self-discrepancy

**DOI:** 10.3389/fpsyg.2026.1904130

**Published:** 2026-07-27

**Authors:** Jacinth J. X. Tan, Bek Wuay Tang

**Affiliations:** School of Social Sciences, Singapore Management University, Singapore, Singapore

**Keywords:** burnout, emotion, engagement, goal facilitation, self-discrepancy

## Abstract

Employees who perceive greater personal goal facilitation through work (PGFW) experience lower burnout and higher work engagement. This research examined whether self-discrepancies and emotional experiences explain these relationships across two cross-sectional studies involving a nationally representative sample of working adults (Study 1; *N* = 889) and a sample of public school teachers (Study 2; *N* = 228) in Singapore. Participants listed personal goals and rated how much their jobs facilitated these goals, alongside measures of self-discrepancy, emotions, burnout, and engagement. Across both samples, higher PGFW predicted lower burnout and greater engagement. These associations were consistently explained by lower ideal self-discrepancy and greater positive emotions, but less consistently explained by ought self-discrepancy and negative emotions. Findings suggest that organizations may reduce burnout and enhance engagement by fostering employees’ sense that their work supports attainment of their ideal self.

## Introduction

Employment is an important means to achieving meaning in life and personal development (Ward and King, 2017). However, overwhelming work responsibilities can hinder our capacity to fulfil other meaningful life goals. In the extreme, it may lead to burnout—a negative psychological syndrome in response to chronic stressors experienced at work, characterized by feelings of emotional exhaustion, having detached relationships with clients and colleagues, and a reduced sense of personal accomplishment (Maslach et al., 2001). A close counterpart is work engagement—a persistent positive state defined by vigor, dedication, and absorption (Schaufeli et al., 2006). In the absence of burnout, employees can feel engaged at work, even when they are expending significant time and effort in their work (Bakker et al., 2023).

Most research has examined how burnout and work engagement are linked to the degree of fit experienced at work, conceptualized as perceived mismatches between job demands and resources (i.e., Bakker et al., 2023), or misalignments between individual traits and the organization (Cable and Edwards, 2004; Leiter and Maslach, 1999). However, much less research has investigated fit with employees’ personal goals. The current research examined whether and how the perception of personal goal facilitation through work (PGFW) predicts burnout and work engagement.

### PGFW as workplace fit

The person-job fit perspective of burnout posits that employee well-being varies not only with job demands, but also whether demands are misaligned with employees’ expectations (Bakker et al., 2023). Therefore, the greater the misalignment, the greater the likelihood of burnout or low work engagement. Such mismatches may occur in any of the six identified domains, namely workload, control, reward, community, fairness, and values (Leiter and Maslach, 1999).

Personal goal facilitation through work (PGFW) refers to “the perception of the extent to which one’s job facilitates the attainment of one’s personal goals” (Doest et al., 2006, p. 192). From the person-job fit perspective, PGFW may be conceptualized as the experienced mismatch or fit between opportunities and resources provided by the work environment and individuals’ personal goals. PGFW is most conceptually aligned to the domain of (mis)match in values—whether the job requires employees to engage in behaviors that are aligned with their personal important beliefs. Personal goals represent outcomes that individuals set to achieve, which can be value-driven (Schwartz, 1992). When making vocational decisions, people indeed consider if a job can fulfil personally meaningful goals (Elias et al., 2018; Ward and King, 2017). Although value mismatch has been found to significantly predict greater burnout and lower work engagement (Dyląg et al., 2013; Kilroy et al., 2017; Veage et al., 2014), there is limited evidence for whether perceived PGFW would predict similar outcomes.

Recent organizational research suggests that workplace resources play an important role in reducing burnout, as well as promoting employee engagement and well-being. For example, high-performance work systems have been shown to reduce burnout directly and indirectly by lowering employees’ perceived job demands while enhancing overall quality of life, highlighting how organizational experiences that support employees’ needs can foster psychological well-being (Dorta-Afonso and Romero-Domínguez, 2025; Liao et al., 2026; Wang et al., 2022). A recent meta-analysis also found that employees who perceive greater personal psychological capacity at work also experience greater work engagement (Goyal et al., 2024). These findings suggest that work characteristics that are seen as facilitating employees’ valued outcomes may be an important, yet underexplored, determinant of burnout and engagement.

So far, only two studies have examined the link between PGFW and burnout among healthcare professionals (Doest et al., 2006; Pisanti et al., 2016). Consistent with the value-fit literature, they found that healthcare professionals who saw their job as significantly facilitating their personal goals (i.e., high in PGFW) reported lower emotional exhaustion, higher personal accomplishment (Doest et al., 2006), and higher work engagement (Pisanti et al., 2016). However, these studies focused on healthcare professionals, limiting generalizability of their findings to other professions. Furthermore, these studies assessed PGFW as *nomothetic* personal goals assumed to be shared by all healthcare professionals, neglecting individual differences in personal goals even in the same profession. Finally, the underlying mechanisms for why perceived PGFW should matter for burnout and work engagement were not examined.

The current research sought to fill these gaps. First, we aimed to establish the association between PGFW—assessed as *idiographic* personal goals uniquely defined by the individual (Wiese and Salmela-Aro, 2008)—burnout and work engagement across occupational groups. The idiographic approach allows for variation in personal goals within the same profession that are personally meaningful to individuals, strengthening the face validity of PGFW. We hypothesized that:

Hypothesis 1: For burnout, PGFW assessed idiographically will be negatively associated with (a) emotional exhaustion, and (b) depersonalization, but positively associated with (c) personal accomplishment.

Hypothesis 2: For work engagement, PGFW assessed idiographically will be positively associated with (a) vigor, (b) dedication, and (c) absorption.

### The role of self-discrepancy and emotions

Second, we examined a potential mechanism through which PGFW predicts burnout and engagement. People experiencing burnout often report a loss of psychological connection to their jobs, which can affect their motivation and self-identity (Maslach and Leiter, 2017). Edwards (1992) has similarly theorized that work stress can emerge from discrepancies between perceived and desired states in personally important domains.

Self-discrepancy theory proposes that individuals hold multiple self-concepts categorized into actual and possible selves (Higgins, 2012). The *actual self* refers to qualities currently possessed. Possible selves include the *ideal self* (attributes one aspires to possess) and the *ought self* (qualities one is expected to possess based on duties or obligations). Inconsistencies between the actual self and possible selves give rise to emotional states that motivate self-discrepancy reduction (Higgins, 2012; Barnett et al., 2017).

Personal goals represent concrete manifestations of individuals’ broader aspirations and desired future selves. Following self-discrepancy theory (Higgins, 2012), discrepancies between one’s actual self and ideal/ought self arise when individuals perceive their insufficient progress toward who they ideally wish or ought to become. Consequently, work experiences that facilitate employees’ personally meaningful goals should signal progress toward these aspirations or duties, thereby reducing perceived discrepancies between the actual and ideal/ought self. Conversely, when work is perceived as hindering progress toward valued goals, employees may experience greater ideal/ought self-discrepancy because they feel that their work is preventing them from becoming the person they aspire or ought to be. Accordingly, we hypothesized that:

Hypothesis 3: PGFW will be negatively associated with both perceived ideal and ought self-discrepancies.

We further expected the effect of PGFW on burnout and engagement to be mediated by dissatisfaction or discomfort elicited by self-discrepancy, conceptualized as reduced positive affect or increased negative affect, respectively. Meta-analytic evidence indicates that self-discrepancy is associated with greater negative affect and lower positive affect generally, regardless of the type of self-discrepancy (Mason et al., 2019). Although most studies on burnout and work engagement have focused on negative affect for burnout (e.g., Leiter and Durup, 1994; Raedeke et al., 2013) and positive affect for work engagement (e.g., Ouweneel et al., 2012; Sharma and Goyal, 2022), this distinction was neither conceptually motivated nor based on discriminant evidence. Since self-discrepancy impacts both types of affect (Mason et al., 2019), we posited that both may be involved in how self-discrepancy explains burnout and engagement, as follows:

Hypothesis 4: The associations between PGFW and burnout/work engagement will be mediated by lower ideal and ought self-discrepancies followed by lower negative affect.

Hypothesis 5: The associations between PGFW and burnout/work engagement will be mediated by lower ideal and ought self-discrepancies followed by higher positive affect.

**Figure 1:**
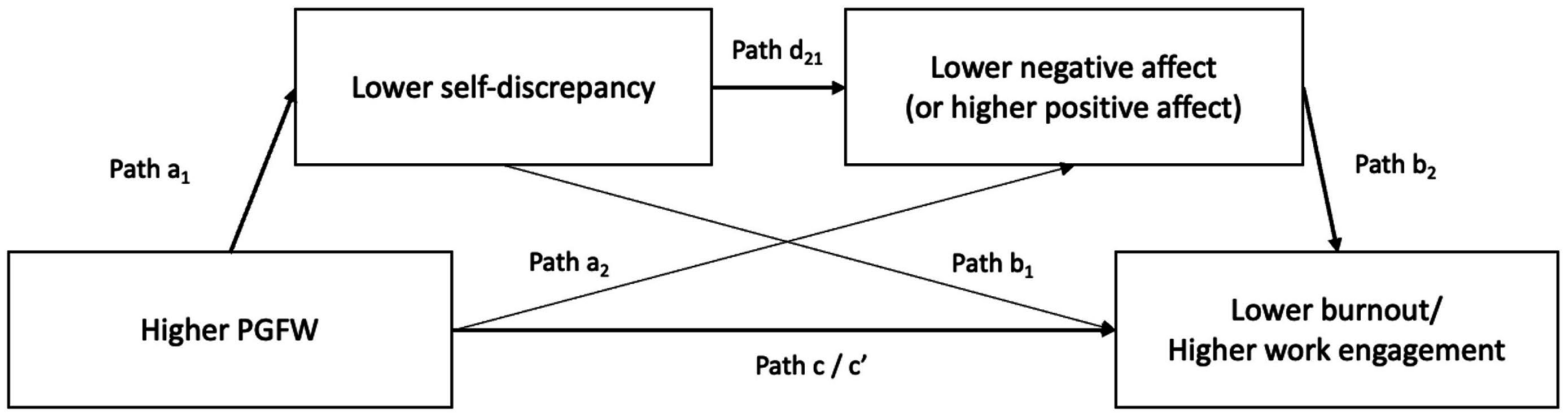
Hypothesized serial mediation by self-discrepancy and affect. Bolded arrows represent hypothesized pathways.

We tested our hypothesized paths (Figure 1) across two cross-sectional studies involving a large nationally representative sample of working adults (Study 1) and a sample of public school teachers (Study 2) in Singapore.

## Study 1

This study sought to provide initial evidence for whether and how idiographically assessed PGFW predicts burnout and work engagement. To enhance generalizability, we studied a nationally representative sample of working adults across occupational groups.

### Method

#### Participants

Nine hundred and five full-time Singaporean working adults were recruited through Qualtrics Online Panel. After removing participants who failed quality checks, the final sample consisted of 889 participants (50% female), with an average age of 42.35 (*SD* = 12.28). Demographics were representative of full-time working adults in Singapore: 82% Chinese, 54% in supervisory roles, and 61% with at least tertiary education.

#### Procedure

Participants answered survey questions online in a fixed order: PGFW, goal importance, self-discrepancy, negative and positive affect, burnout, work engagement, and demographics. All measures have demonstrated good construct validity in previous research.

#### Key measures

##### PGFW

This was assessed by asking participants to evaluate the extent to which their work facilitated the attainment of their personal goals through an idiographic approach adapted from Wiese and Salmela-Aro (2008). Participants listed three important personal goals. For each, they rated the question “To what extent can you achieve the goal of (goal) through your current work/job?” on a 5-point Likert scale (1 = *to a very limited extent*, 5 = *to a very great extent*). Responses were averaged (*α* = 0.70) with higher scores reflecting greater PGFW.

##### Self-Discrepancy

Adapted from the Integrated Self-Discrepancy Index (Hardin and Lakin, 2009), participants listed five attributes for their ideal and ought selves, and rated how much they applied currently on a 5-point scale (1 = *completely applies to me*, 5 = *does not apply to me at all*). Higher scores reflect higher ideal discrepancy (*α* = 0.87) and ought discrepancy (α = 0.84).

##### Negative and positive affect

Participants rated items from the Positive and Negative Affect Schedule (PANAS; Watson et al., 1988) on a 5-point scale. Two affect scores were computed by averaging items that captured negative affect (*α* = 0.93) and positive affect (*α* = 0.93), with higher scores reflecting stronger affect.

##### Burnout

Participants completed the Maslach Burnout Inventory-General Survey (Maslach et al., 2017; Wang et al., 2024) on a 7-point scale (0 = *never*, 6 = *every day*). Three dimensions scores were computed by averaging items that captured exhaustion (*α* = 0.93), cynicism (*α* = 0.85), and professional efficacy (*α* = 0.85), with higher scores reflecting stronger feelings on each dimension.

##### Work engagement

Participants completed the 9-item Utrecht Work Engagement Scale (UWES; Schaufeli et al., 2006) on a 7-point scale (0 = *never*, 6 = *every day*). Three dimensions scores were computed by averaging items that captured vigor (*α* = 0.87), dedication (*α* = 0.93), and absorption (*α* = 0.81).

### Results

The means, standard deviations and zero-order correlations involving all study variables are presented in Table 1. The observed relationships among PGFW, self-discrepancy, affect, burnout, and work engagement were consistent with theoretical expectations.

**Table 1:** Means, standard deviations, and correlations for studies 1 and 2.

Variable	Study 1 *M* (*SD*)	Study 2 *M* (*SD*)	1	2	3	4	5	6	7	8	9	10	11
1. PGFW	2.99 (0.94)	2.95 (0.82)	—	−0.20***	−0.19***	0.22***	0.35***	0.36***	0.31***	−0.39***	−0.28**	0.42***	−0.04
2. Emotional exhaustion	2.96 (1.69)	4.31 (1.17)	−0.28***	—	0.69***	−0.02	−0.34***	−0.38***	−0.26***	0.22***	0.14**	−0.33***	0.52***
3. Cynicism/Depersonalization	2.98 (1.56)	3.03 (1.05)	−0.24***	0.39***	—	−0.01	−0.35***	−0.40***	−0.25***	0.17***	0.12**	−0.34***	0.53***
4. Professional Efficacy/Personal accomplishment	4.07 (1.25)	4.64 (0.74)	0.30***	−0.21**	−0.23**	—	0.55***	0.53***	0.52***	−0.24***	−0.22**	0.49***	−0.19***
5. Vigor	3.28 (1.69)	3.42 (1.24)	0.39***	−0.51***	−0.33***	0.58***	—	0.80***	0.75***	−0.40***	−0.32**	0.69***	−0.23***
6. Dedication	3.52 (1.84)	4.73 (1.19)	0.31***	−0.38***	−0.37***	0.60***	0.67***	—	0.76***	−0.42***	−0.30**	0.71***	−0.23***
7. Absorption	3.60 (1.59)	4.31 (1.08)	0.33***	−0.20**	−0.22***	0.50***	0.63***	0.66***	—	−0.31***	−0.24**	0.59***	−0.17***
8. Ideal self-discrepancy	2.90 (0.96)	3.66 (1.24)	−0.22***	0.25***	0.18**	−0.32***	−0.37***	−0.34***	−0.36***	—	0.64**	−0.48***	0.10**
9. Ought self-discrepancy	2.64 (0.88)	3.55 (1.48)	−0.16*	0.23***	0.20**	−0.22***	−0.21**	−0.19**	−0.15*	0.54***	—	−0.38***	0.14***
10. Positive affect	2.26 (0.88)	2.17 (0.72)	0.35***	−0.44***	−0.31***	0.57***	0.68***	0.74***	0.57***	−0.35***	−0.17**	—	−0.06
11. Negative affect	3.17 (0.88)	3.18 (0.68)	−0.18**	0.55***	0.31***	−0.17**	−0.28***	−0.10	−0.09	0.26***	0.24***	−0.16*	—

*p < 0.05, **p < 0.01, ***p < 0.001. Correlations for Study 1 are presented above the diagonal and Study 2 below the diagonal.

### Burnout and work engagement

Controlling for age and gender, higher PGFW significantly predicted lower exhaustion (*b* = −0.37), lower cynicism (*b* = −0.33), and higher professional efficacy (*b* = 0.30), supporting Hypothesis 1. Higher PGFW also significantly predicted higher vigor (*b* = 0.65), higher dedication (*b* = 0.72), and higher absorption (*b* = 0.54), supporting Hypothesis 2.

### Self-discrepancy

Higher PGFW was significantly negatively associated with lower ideal self-discrepancy (*b* = −0.40) and lower ought self-discrepancy (*b* = −0.27), supporting Hypothesis 3.

### Serial mediation via self-discrepancy and negative affect

Mediation was assessed using R PROCESS Macro Model 6 (5,000 bootstrap samples). In the model involving ideal self-discrepancy and burnout, higher PGFW predicted lower ideal discrepancy (*b* = −0.40), which in turn predicted lower negative affect (*b* = 0.09), followed by less burnout (*b* = 0.97 for exhaustion, *b* = 0.92 for cynicism, *b* = −0.24 for professional efficacy). The indirect effect of ideal self-discrepancy and negative affect was significant (*b* = −0.04 for exhaustion, *b* = −0.03 for cynicism, *b* = 0.01 for professional efficacy).

In the model involving ideal self-discrepancy and work engagement, higher PGFW similarly predicted lower ideal discrepancy (*b* = −0.40), which in turn predicted lower negative affect (*b* = 0.09), followed by greater engagement (*b* = −0.36 for vigor, *b* = −0.39 for dedication, *b* = −0.25 for absorption). The indirect effect of ideal self-discrepancy and negative affect was significant (all *b*s = 0.01 for vigor, dedication and absorption).

Nonetheless, the direct effects (path c′) of PGFW on all burnout and engagement dimensions remained significant, suggesting partial mediation and partial support for Hypothesis 4.

The model involving ought self-discrepancy largely mirrored that of ideal self-discrepancy, suggesting that the mediating role of self-discrepancy was similar regardless of the type of discrepancy.

### Serial mediation via self-discrepancy and positive affect

In the model involving ideal self-discrepancy and burnout, higher PGFW predicted lower ideal discrepancy (*b* = −0.40), which in turn predicted higher positive affect (*b* = −0.34), followed by less burnout (*b* = −0.54 for exhaustion, *b* = −0.57 for cynicism, *b* = 0.68 for professional efficacy). The indirect effect of ideal self-discrepancy and positive affect was significant (*b* = −0.07 for exhaustion, *b* = −0.08 for cynicism, *b* = 0.09 for professional efficacy).

In the model involving ideal self-discrepancy and work engagement, higher PGFW similarly predicted lower ideal discrepancy (*b* = −0.40), which in turn predicted higher positive affect (*b* = −0.34), followed by greater engagement (*b* = 1.20 for vigor, *b* = 1.36 for dedication, *b* = 1.00 for absorption). As well, the indirect effect of ideal self-discrepancy and positive affect was significant (*b* = 0.16 for vigor, *b* = 0.19 for dedication, *b* = 0.14 for absorption).

Importantly, the direct effects (path c′) of PGFW on all burnout and work engagement dimensions became non-significant, suggesting full mediation and support for Hypothesis.

The model involving ought self-discrepancy largely mirrored that of ideal self-discrepancy, except that there was only evidence of partial mediation for the engagement dimensions. All indirect effects central to Hypotheses 4 and 5 are summarized in Table 2.

**Table 2:** Bootstrapped indirect effects of PGFW on burnout and work engagement among general working adults in Study 1.

Outcome (Path from PGFW)	Ideal self-discrepancy	Ought self-discrepancy	Positive affect	Negative affect	Ideal → Positive affect	Ideal → Negative affect	Ought → Positive affect	Ought → Negative affect
Exhaustion	**−0.08** [−0.13, −0.03]	−0.01 [−0.05, 0.02]	**−0.14** [−0.19, −0.09]	0.003 [−0.07, 0.07]	**−0.07** [−0.10, −0.05]	**−0.04** [−0.06, −0.01]	**−0.07** [−0.10, −0.05]	**−0.04** [−0.06, −0.02]
Cynicism	−0.04 [−0.08, 0.001]	−0.002 [−0.03, 0.03]	**−0.15** [−0.20, −0.10]	0.003 [−0.07, 0.06]	**−0.08** [−0.11, −0.05]	**−0.03** [−0.06, −0.01]	**−0.04** [−0.07, −0.03]	**−0.03** [−0.06, −0.02]
Professional Efficacy	**0.08** [0.04, 0.12]	**0.06** [0.03, 0.09]	**0.17** [0.12, 0.23]	0.001 [−0.02, 0.02]	**0.09** [0.07, 0.12]	**0.01** [0.002, 0.02]	**0.05** [0.03, 0.07]	**0.01** [0.003, 0.02]
Vigor	**0.20** [0.15, 0.26]	**0.11** [0.07, 0.16]	**0.31** [0.23, 0.39]	0.001 [−0.03, 0.03]	**0.16** [0.12, 0.21]	**0.01** [0.004, 0.03]	**0.09** [0.06, 0.13]	**0.01** [0.01, 0.02]
Dedication	**0.24** [0.17, 0.31]	**0.11** [0.06, 0.16]	**0.35** [0.26, 0.44]	0.01 [−0.03, 0.03]	**0.19** [0.14, 0.24]	**0.01** [0.004, 0.03]	**0.11** [0.07, 0.15]	**0.01** [0.01, 0.03]
Absorption	**0.14** [0.09, 0.20]	**0.07** [0.04, 0.11]	**0.25** [0.19, 0.32]	0.01 [−0.02, 0.02]	**0.14** [0.10, 0.17]	**0.01** [0.002, 0.02]	**0.08** [0.05, 0.11]	**0.01** [0.003, 0.02]

Values are standardized indirect effects with 95% Confidence Intervals estimated from 5,000 bootstrap samples. Significant indirect effects are shown in bold. Full Process Model 6 output is provided in Supplementary Tables S1–S4.

### Discussion

Study 1 found initial evidence that idiographically defined PGFW significantly predicts burnout and work engagement. There was also support for the mechanistic role of self-discrepancy—regardless of type—and affect, although the patterns were more consistent with positive affect than negative affect.

## Study 2

Study 2 focused on public school teachers in Singapore—a relatively homogeneous professional context with extensive work hours compared to global average (Organisation for Economic Co-operation and Development, 2024). This study sought to replicate Study 1’s findings in this population at high burnout risk.

### Method

#### Participants

Two hundred and thirty-five school teachers in Singapore were recruited via email invitations through schools, personal teacher contacts, and snowball sampling. Following data quality checks, the final sample consisted of 228 participants (72% female), with an average age of 34.3 (*SD* = 5.54). Participants had an average of 8.93 years of teaching experience (*SD* = 4.85). The sample was 93% Chinese, and 90% held a bachelor’s degree or higher.

#### Procedure

After providing consent, participants were exposed to a brief self-affirmation manipulation, and then answered questions assessing the same key variables as in Study 1. The self-affirmation manipulation was included to examine whether affirming important personal values could reduce perceived self-discrepancy. Preliminary analyses indicated that the manipulation did not significantly affect self-discrepancy, PGFW, burnout, work engagement, or the hypothesized relationships among these variables (all *ps* > 0.05; results presented in Supplemental material). Accordingly, participants from both conditions were pooled for the primary analyses.

#### Key measures

##### PGFW

The same measure in Study 1 was used.

##### Self-discrepancy

Using an adapted self-expansion version of the Inclusion of Other in the Self scale (Aron et al., 2022), participants selected from seven diagrams showing varying extents of overlap between circles representing their actual self and their ideal/ought self (1 = *no overlap*, 7 = *greatest overlap*). Responses were reverse-coded so higher scores represented higher discrepancy. The use of a seven-point response scale, instead of a five-point scale as in Study 1, followed the response format of the validated measure. Since each study was analyzed independently and our hypotheses focused on within-study associations rather than between-study mean comparisons, this difference is unlikely to affect the interpretation of the findings.

##### Burnout

Participants answered the Maslach Burnout Inventory-Educators Survey (Maslach et al., 2017) on a 7-point intensity scale (1 = *very mild, barely noticeable*, 7 = *very strong, major*). Dimensions included emotional exhaustion (*α* = 0.92), depersonalization (instead of cynicism; *α* = 0.68), and personal accomplishment (instead of professional efficacy; *α* = 0.77).

##### Work engagement

Participants answered the same 9-item UWES as in Study 1 (Schaufeli et al., 2006) that captured vigor (*α* = 0.88), dedication (*α* = 0.69), and absorption (*α* = 0.88).

##### Negative and positive affect

Participants answered the PANAS items as in Study 1 that captured negative affect (*α* = 0.88) and positive affect (*α* = 0.90).

### Results

The means, standard deviations and zero-order correlations involving all study variables are also presented in Table 1. Again, the observed relationships among PGFW, self-discrepancy, affect, burnout, and work engagement were consistent with theoretical expectations.

### Burnout and work engagement

Controlling for age and gender, higher PGFW significantly predicted lower exhaustion (*b* = −0.40), lower depersonalization (*b* = −0.32), and higher personal accomplishment (*b* = 0.27), supporting Hypothesis 1. Higher PGFW also significantly predicted higher vigor (*b* = 0.59), dedication (*b* = 0.47), and absorption (*b* = 0.44), supporting Hypothesis 2.

### Self-discrepancy

Higher PGFW was significantly negatively associated with ideal self-discrepancy (*b* = −0.34) and ought self-discrepancy (*b* = −0.30), supporting Hypothesis 3.

### Serial mediation via ideal self-discrepancy and negative affect

Similar mediation tests were conducted as in Study 1. In the model involving ideal self-discrepancy and burnout, higher PGFW predicted lower ideal discrepancy (*b* = −0.34), which in turn predicted lower negative affect (*b* = 0.14), followed by less exhaustion (*b* = 0.82) and depersonalization (*b* = 0.38). The indirect effect of ideal self-discrepancy and negative affect was significant for both exhaustion (*b* = −0.04) and depersonalization (*b* = −0.02). These patterns were not observed for professional efficacy.

In the model involving ideal self-discrepancy and work engagement, higher PGFW similarly predicted lower ideal discrepancy (*b* = −0.34), which in turn predicted lower negative affect (*b* = 0.14), followed by greater vigor (*b* = −0.28). The indirect effect of ideal self-discrepancy and negative affect was also significant for vigor (*b* = 0.01). These patterns were not seen for dedication and absorption.

Furthermore, the direct effects (path c′) of PGFW on exhaustion, depersonalization and vigor remained significant, suggesting partial mediation and partial support for Hypothesis 4.

The model with ought self-discrepancy also mirrored that of ideal self-discrepancy, indicating similar mediating role of self-discrepancy across types.

### Serial mediation via self-discrepancy and positive affect

In the model involving ideal self-discrepancy and burnout, higher PGFW predicted lower ideal discrepancy (*b* = −0.34), which in turn predicted higher positive affect (*b* = −0.16), followed by less burnout (*b* = −0.61 for exhaustion, *b* = −0.37 for depersonalization, *b* = 0.53 for personal accomplishment). The indirect effect of ideal self-discrepancy and positive affect was also significant (*b* = −0.03 for exhaustion, *b* = −0.02 for depersonalization, *b* = 0.03 for personal accomplishment).

In the model involving ideal self-discrepancy and work engagement, higher PGFW similarly predicted lower ideal discrepancy (*b* = −0.40), which in turn predicted higher positive affect (*b* = −0.16), followed by greater engagement (*b* = 1.06 for vigor, *b* = 1.21 for dedication, *b* = 0.73 for absorption). As well, the indirect effect of ideal self-discrepancy and positive affect was significant (*b* = 0.05 for vigor, *b* = 0.06 for dedication, *b* = 0.04 for absorption).

Importantly, the direct effects (path c′) of PGFW on all burnout dimensions and dedication became non-significant, suggesting full mediation and support for Hypothesis 5 largely for burnout.

Unexpectedly, the serial mediation pathway via ought self-discrepancy and positive affect was non-significant for all burnout and engagement dimensions. All indirect effects central to Hypotheses 4 and 5 are summarized in Table 3.

**Table 3:** Bootstrapped indirect effects of PGFW on burnout and work engagement among teachers in Study 2.

Outcome (Path from PGFW)	Ideal self-discrepancy	Ought self-discrepancy	Positive affect	Negative affect	Ideal → Positive affect	Ideal → Negative affect	Ought → Positive affect	Ought → Negative affect
Exhaustion	−0.03 [−0.07, 0.01]	−0.02 [−0.06, 0.01]	**−0.14** [−0.23, −0.07]	−0.09 [−0.18, 0.01]	**−0.03** [−0.06, −0.01]	**−0.04** [−0.08, −0.01]	−0.01 [−0.03, 0.001]	**−0.02** [−0.05, −0.004]
Depersonalization	−0.02 [−0.07, 0.02]	−0.02 [−0.06, 0.0004]	**−0.09** [−0.16, −0.03]	−0.04 [−0.09, 0.004]	**−0.02** [−0.04, −0.005]	**−0.02** [−0.04, −0.003]	−0.01 [−0.02, 0.001]	**−0.01** [−0.02, −0.002]
Personal accomplishment	**0.05** [0.02, 0.09]	**0.02** [0.002, 0.05]	**0.12** [0.07, 0.19]	0.01 [−0.01, 0.03]	**0.03** [0.01, 0.05]	0.003 [−0.003, 0.01]	0.01 [−0.001, 0.02]	0.003 [−0.001, 0.01]
Vigor	**0.01** [0.001, 0.03]	0.03 [−0.002, 0.07]	**0.24** [0.14, 0.36]	0.03 [−0.004, 0.08]	**0.05** [0.02, 0.10]	**0.01** [0.001, 0.03]	0.02 [−0.002, 0.05]	**0.01** [0.001, 0.02]
Dedication	−0.002 [−0.01, 0.01]	0.03 [−0.0001, 0.08]	**0.28** [0.16, 0.42]	−0.004 [−0.04, 0.02]	**0.06** [0.02, 0.11]	−0.002 [−0.01, 0.01]	0.02 [−0.002, 0.05]	0.001 [−0.01, 0.01]
Absorption	−0.003 [−0.02, 0.01]	0.02 [−0.004, 0.06]	**0.17** [0.09, 0.26]	−0.01 [0.02, 0.01]	**0.04** [0.01, 0.07]	−0.003 [−0.02, 0.01]	0.01 [−0.001, 0.03]	0.003 [−0.01, 0.01]

Values are standardized indirect effects with 95% Confidence Intervals estimated from 5,000 bootstrap samples. Significant indirect effects are shown in bold. Full process Model 6 output is provided in Supplementary Tables S5–S8.

### Discussion

Study 2 replicated the some of the key findings of Study 1 in a sample of public school teachers—an occupational group characterized by relatively high burnout risk. While some indirect effects involving ought self-discrepancy and negative affect emerged for selected outcomes, the pathways involving ideal self-discrepancy and positive affect were most consistent.

## General discussion

This research established that idiographic PGFW consistently predicted burnout, work engagement, and self-discrepancies across diverse and specialized occupational settings. Additionally, ideal self-discrepancy and positive affect most consistently and fully explained the association between PGFW and employee well-being.

The present findings advance the literature in several ways. First, they extend research on personal goal facilitation through work (PGFW) by demonstrating that its effects on burnout and work engagement generalize across both a nationally representative workforce and a profession characterized by high burnout risk. Second, the findings extend self-discrepancy theory (Higgins, 2012) by identifying workplace experiences as an important antecedent of employees’ perceived self-discrepancy. Specifically, we demonstrate that employees’ perceptions that their work facilitates personally meaningful goals can reduce discrepancies between their actual and ideal selves, thereby improving well-being. The findings also contribute to the burnout and work engagement literature by identifying a motivational and self-evaluative mechanism that complements existing perspectives emphasizing job demands and resources (Bakker et al., 2023). Specifically, our results suggest that employees’ evaluations of whether work enables progress toward their personally valued aspirations constitute an important psychological resource that promotes engagement and reduces burnout through reducing ideal self-discrepancy and fostering positive affect.

Practically, these findings suggest that organizations may reduce burnout and enhance work engagement by fostering employees’ perceptions that their work facilitates personally meaningful goals. Instead of focusing solely on reducing job demands, organizations may benefit from creating work environments that enable employees to make meaningful progress toward their personal aspirations. For example, managers can better understand employees’ personal goals through regular career and development discussions and frame day-to-day work tasks in ways that support these broader aspirations. Organizational practices such as job crafting opportunities, individualized career development plans and mentoring programs, may further strengthen employees’ perceptions that their work contributes to their ideal selves. Because our findings suggest that these benefits operate primarily by reducing ideal self-discrepancy and promoting positive emotions, organizations should consider employee development not only in terms of improving job performance, but also as a means of helping employees perceive greater alignment between their work and their personally valued goals.

We note several limitations of the current work. First, because both studies employed cross-sectional designs and self-reports, the proposed mediation pathways cannot be interpreted as causal and may be susceptible to common method variance. Although the observed relationships were theoretically consistent and replicated across two independent samples, future research should employ longitudinal, experience-sampling or experimental designs to strengthen causal inference, as well as include behavioral or objective measures (e.g., cardiovascular measures to assess burnout or engagement; Ganster et al., 2018) to reduce potential method bias. In addition, the depersonalization subscale in Study 2 demonstrated slightly lower reliability (*α* = 0.68), so findings involving this outcome should be interpreted cautiously. Second, our measure of PGFW does not distinguish between work-related and non-work-related personal goals. Examining whether facilitation of different types of goals differentially influences self-discrepancy and well-being may further refine theories of person-job fit and help organizations tailor interventions more effectively. Third, the self-discrepancy measure used different response formats across the two studies, although the consistent pattern of findings suggests that the observed relationships were robust across measurement formats. Nevertheless, future research would benefit from adopting a common response scale to facilitate direct comparisons across samples.

Building on these limitations, future research should examine how changes in PGFW and self-discrepancy unfold over time to predict emotional experiences, burnout, and work engagement. Experimental and intervention studies may further test whether organizational initiatives that enhance employees’ perceptions of personal goal facilitation, or psychological interventions that help employees reappraise work situations to reduce self-discrepancies, lead to sustained improvements in employee well-being.

Finally, although the present research identified ideal self-discrepancy and positive affect as key explanatory mechanisms, future research should investigate additional conditions under which these relationships are strengthened or weakened. For example, individual differences such as regulatory focus (Lanaj et al., 2012) or growth mindsets (Murphy and Reeves, 2020) may influence the extent to which employees perceive progress toward their ideal selves through work. Likewise, organizational characteristics—including job autonomy, leadership style, and perceived organizational support—and broader cultural contexts that differ in the value placed on personal aspirations versus social obligations may moderate the effects of PGFW on self-discrepancy and employee well-being (Tang et al., 2023). Examining these contextual and individual factors would further refine our understanding of when and for whom personal goal facilitation through work is most beneficial.

## Data Availability

The datasets presented in this study can be found in online repositories. The names of the repository/repositories and accession number(s) can be found at: https://osf.io/5fua2/overview?view_only=fac3f50169e34a9fb4b526053f2da377.
