# Supplementary material for: Personal goal facilitation, burnout and work engagement: the role of self-discrepancy
Source: Front Psychol. 2026 Jul 27;17:1904130. doi: 10.3389/fpsyg.2026.1904130 (PMC13453788; doi:10.3389/fpsyg.2026.1904130)
Supplement: Supplementary file 1 [file Table_1.DOCX]

**Supplemental Material**

**Table S1**
*Results from Serial Mediation Analyses via Ideal Self-Discrepancy and Negative Affect in Study 1*

| Pathway | *b* | *SE* | *95% CI* |  |  |  |  |  |  |
| --- | --- | --- | --- | --- | --- | --- | --- | --- | --- |
| *X* (PGFW); *M_1_* (Ideal self-discrepancy); *M_2_* (Negative affect) | | | | | | | | | |
| Effect of X on M_1_ (Path a_1_) | -0.40 | 0.03 | [-0.46, -0.33] |  |  |  |  |  |  |
| Effect of X on M_2_ (Path a_2_) | -0.003 | 0.04 | [-0.07, 0.07] |  |  |  |  |  |  |
| Effect of M_1_ on M_2_ (Path d_21_) | 0.09 | 0.03 | [0.03, 0.16] |  |  |  |  |  |  |
|  | *Y* (Exhaustion) | | | *Y* (Cynicism) | | | *Y* (Professional efficacy) | | |
|  | *b* | *SE* | *95% CI* | *b* | *SE* | *95% CI* | *b* | *SE* | *95% CI* |
| Direct Effect of X on Y (Path c’) | -0.23 | 0.06 | [-0.35, -0.12] | -0.23 | 0.05 | [-0.34, -0.12] | 0.20 | 0.05 | [0.10, 0.30] |
| Direct Effect of M_1_ on Y (Path b_1_) | 0.20 | 0.06 | [0.09, 0.31] | 0.10 | 0.05 | [-0.004, 0.20] | -0.21 | 0.05 | [-0.30, -0.12] |
| Direct Effect of M_2_ on Y (Path b_2_) | 0.97 | 0.05 | [0.87, 1.07] | 0.92 | 0.05 | [0.82, 1.01] | -0.24 | 0.05 | [-0.33, -0.16] |
| Total effect of X on Y | -0.35 | 0.06 | [-0.47, -0.24] | -0.31 | 0.05 | [-0.42, -0.20] | 0.29 | 0.04 | [0.21, 0.38] |
| Indirect effect of X on Y via M_1_ | -0.08 | 0.02 | [-0.13, -0.03] | -0.04 | 0.02 | [-0.08, 0.001] | 0.08 | 0.02 | [0.04, 0.12] |
| Indirect effect of X on Y via M_2_ | 0.003 | 0.04 | [-0.07, 0.07] | 0.003 | 0.03 | [-0.07, 0.06] | 0.001 | 0.01 | [-0.02, 0.02] |
| Indirect effect of X on Y via M_1_ and M_2_ | -0.04 | 0.01 | [-0.06, -0.01] | -0.03 | 0.01 | [-0.06, -0.01] | 0.01 | 0.004 | [0.002, 0.02] |

*Continued*

|  | *Y* (Vigor) | | | *Y* (Dedication) | | | *Y* (Absorption) | | |
| --- | --- | --- | --- | --- | --- | --- | --- | --- | --- |
|  | *b* | *SE* | *95% CI* | *b* | *SE* | *95% CI* | *b* | *SE* | *95% CI* |
| Direct Effect of X on Y (Path c’) | 0.42 | 0.06 | [0.30, 0.54] | 0.46 | 0.07 | [0.32, 0.59] | 0.38 | 0.06 | [0.26, 0.50] |
| Direct Effect of M_1_ on Y (Path b_1_) | -0.51 | 0.06 | [-0.62, -0.39] | -0.60 | 0.06 | [-0.72, -0.47] | -0.35 | 0.06 | [-0.47, -0.24] |
| Direct Effect of M_2_ on Y (Path b_2_) | -0.36 | 0.06 | [-0.48, -0.24] | -0.39 | 0.06 | [-0.51, -0.27] | -0.25 | 0.06 | [-0.36, -0.14] |
| Total effect of X on Y | 0.64 | 0.06 | [0.53, 0.75] | 0.71 | 0.06 | [0.59, 0.83] | 0.53 | 0.05 | [0.43, 0.64] |
| Indirect effect of X on Y via M_1_ | 0.20 | 0.03 | [0.15, 0.26] | 0.24 | 0.03 | [0.17, 0.31] | 0.14 | 0.03 | [0.09, 0.20] |
| Indirect effect of X on Y via M_2_ | 0.001 | 0.01 | [-0.03, 0.03] | 0.001 | 0.01 | [-0.03, 0.03] | 0.001 | 0.01 | [-0.02, 0.02] |
| Indirect effect of X on Y via M_1_ and M_2_ | 0.01 | 0.01 | [0.004, 0.03] | 0.01 | 0.01 | [0.004, 0.03] | 0.01 | 0.004 | [0.002, 0.02] |

*Note.* All reported effects were based on 5000 bootstrapping samples.

**Table S2**
*Results from Serial Mediation Analyses via Ought Self-Discrepancy and Negative Affect in Study 1*

| Pathway | *b* | *SE* | *95% CI* |  |  |  |  |  |  |
| --- | --- | --- | --- | --- | --- | --- | --- | --- | --- |
| *X* (PGFW); *M_1_* (Ought self-discrepancy); *M_2_* (Negative affect) | | | | | | | | | |
| Effect of X on M_1_ (Path a_1_) | -0.27 | 0.03 | [-0.33, -0.21] |  |  |  |  |  |  |
| Effect of X on M_2_ (Path a_2_) | -0.004 | 0.04 | [-0.07, 0.07] |  |  |  |  |  |  |
| Effect of M_1_ on M_2_ (Path d_21_ ) | 0.13 | 0.04 | [0.06, 0.21] |  |  |  |  |  |  |
|  | *Y* (Exhaustion) | | | *Y* (Cynicism) | | | *Y* (Professional efficacy) | | |
|  | *b* | *SE* | *95% CI* | *b* | *SE* | *95% CI* | *b* | *SE* | *95% CI* |
| Direct Effect of X on Y (Path c’) | -0.30 | 0.06 | [-0.41, -0.19] | -0.27 | 0.05 | [-0.37, -0.17] | 0.23 | 0.05 | [0.13, 0.32] |
| Direct Effect of M_1_ on Y (Path b_1_) | 0.05 | 0.06 | [-0.06, 0.17] | 0.01 | 0.05 | [-0.10, 0.11] | -0.21 | 0.05 | [-0.32, -0.11] |
| Direct Effect of M_2_ on Y (Path b_2_) | 0.98 | 0.05 | [0.88, 1.09] | 0.92 | 0.05 | [0.83, 1.02] | -0.24 | 0.05 | [-0.33, -0.15] |
| Total effect of X on Y | -0.35 | 0.06 | [-0.47, -0.24] | -0.31 | 0.05 | [-0.42, -0.20] | 0.29 | 0.04 | [0.21, 0.38] |
| Indirect effect of X on Y via M_1_ | -0.01 | 0.02 | [-0.05, 0.02] | -0.002 | 0.01 | [-0.03, 0.03] | **0.06** | **0.02** | **[0.03, 0.09]** |
| Indirect effect of X on Y via M_2_ | -0.004 | 0.04 | [-0.07, 0.07] | -0.004 | 0.03 | [-0.07, 0.06] | 0.001 | 0.01 | [-0.02, 0.02] |
| Indirect effect of X on Y via M_1_ and M_2_ | -0.04 | 0.01 | [-0.06, -0.02] | -0.03 | 0.01 | [-0.06, -0.02] | 0.01 | 0.003 | [0.003, 0.02] |

*Continued*

|  | *Y* (Vigor) | | | *Y* (Dedication) | | | *Y* (Absorption) | | |
| --- | --- | --- | --- | --- | --- | --- | --- | --- | --- |
|  | *b* | *SE* | *95% CI* | *b* | *SE* | *95% CI* | *b* | *SE* | *95% CI* |
| Direct Effect of X on Y (Path c’) | 0.51 | 0.06 | [0.40, 0.63] | 0.58 | 0.07 | [0.46, 0.71] | 0.45 | 0.06 | [0.33, 0.57] |
| Direct Effect of M_1_ on Y (Path b_1_) | -0.41 | 0.07 | [-0.54, -0.28] | -0.40 | 0.07 | [-0.55, -0.26] | -0.27 | 0.06 | [-0.40, -0.15] |
| Direct Effect of M_2_ on Y (Path b_2_) | -0.36 | 0.06 | [-0.49, -0.24] | -0.40 | 0.07 | [-0.53, -0.28] | -0.25 | 0.06 | [-0.36, -0.14] |
| Total effect of X on Y | 0.64 | 0.06 | [0.53, 0.75] | 0.71 | 0.06 | [0.59, 0.83] | 0.53 | 0.05 | [0.42, 0.64] |
| Indirect effect of X on Y via M_1_ | 0.11 | 0.02 | [0.07, 0.16] | 0.11 | 0.02 | [0.06, 0.16] | 0.07 | 0.02 | [0.04, 0.11] |
| Indirect effect of X on Y via M_2_ | 0.002 | 0.01 | [-0.02, 0.03] | 0.002 | 0.01 | [-0.03, 0.03] | 0.001 | 0.01 | [-0.02, 0.02] |
| Indirect effect of X on Y via M_1_ and M_2_ | 0.01 | 0.005 | [0.01, 0.02] | 0.01 | 0.01 | [0.01, 0.03] | 0.01 | 0.004 | [0.003, 0.02] |

*Note.* All reported effects were based on 5000 bootstrapping samples.

**Table S3**
*Results from Serial Mediation Analyses via Ideal Self-Discrepancy and Positive Affect in Study 1*

| Pathway | *b* | *SE* | *95% CI* |  |  |  |  |  |  |
| --- | --- | --- | --- | --- | --- | --- | --- | --- | --- |
| *X* (PGFW); *M_1_* (Ideal self-discrepancy); *M_2_* (Positive affect) | | | | | | | | | |
| Effect of X on M_1_ (Path a_1_) | -0.40 | 0.03 | [-0.46, -0.33] |  |  |  |  |  |  |
| Effect of X on M_2_ (Path a_2_) | 0.26 | 0.03 | [0.19, 0.32] |  |  |  |  |  |  |
| Effect of M_1_ on M_2_ (Path d_21_ ) | -0.34 | 0.03 | [-0.40, -0.28] |  |  |  |  |  |  |
|  | *Y* (Exhaustion) | | | *Y* (Cynicism) | | | *Y* (Professional efficacy) | | |
|  | *b* | *SE* | *95% CI* | *b* | *SE* | *95% CI* | *b* | *SE* | *95% CI* |
| Direct Effect of X on Y (Path c’) | -0.10 | 0.07 | [-0.23, 0.04] | -0.09 | 0.06 | [-0.21, 0.04] | 0.03 | 0.05 | [-0.07, 0.12] |
| Direct Effect of M_1_ on Y (Path b_1_) | 0.10 | 0.07 | [-0.04, 0.24] | -0.01 | 0.07 | [-0.14, 0.12] | -0.002 | 0.05 | [-0.09, 0.09] |
| Direct Effect of M_2_ on Y (Path b_2_) | -0.54 | 0.08 | [-0.70, -0.38] | -0.57 | 0.07 | [-0.71, -0.44] | 0.68 | 0.05 | [0.58, 0.78] |
| Total effect of X on Y | -0.35 | 0.06 | [-0.47, -0.24] | -0.31 | 0.05 | [-0.42, -0.20] | 0.29 | 0.04 | [0.21, 0.38] |
| Indirect effect of X on Y via M_1_ | -0.04 | 0.03 | [-0.10, 0.01] | 0.005 | 0.03 | [-0.05, 0.06] | 0.001 | 0.02 | [-0.04, 0.04] |
| Indirect effect of X on Y via M_2_ | -0.14 | 0.03 | [-0.19, -0.09] | -0.15 | 0.03 | [-0.20, -0.10] | 0.17 | 0.03 | [0.12, 0.23] |
| Indirect effect of X on Y via M_1_ and M_2_ | -0.07 | 0.01 | [-0.10, -0.05] | -0.08 | 0.01 | [-0.11, -0.05] | 0.09 | 0.01 | [0.07, 0.12] |

*Continued*

|  | *Y* (Vigor) | | | *Y* (Dedication) | | | *Y* (Absorption) | | |
| --- | --- | --- | --- | --- | --- | --- | --- | --- | --- |
|  | *b* | *SE* | *95% CI* | *b* | *SE* | *95% CI* | *b* | *SE* | *95% CI* |
| Direct Effect of X on Y (Path c’) | 0.12 | 0.05 | [0.01, 0.22] | 0.11 | 0.06 | [-0.001, 0.22] | 0.13 | 0.06 | [0.02, 0.24] |
| Direct Effect of M_1_ on Y (Path b_1_) | -0.13 | 0.05 | [-0.23, -0.03] | -0.17 | 0.06 | [-0.29, -0.05] | -0.04 | 0.06 | [-0.15, 0.08] |
| Direct Effect of M_2_ on Y (Path b_2_) | 1.20 | 0.05 | [1.10, 1.31] | 1.36 | 0.06 | [1.25, 1.48] | 1.00 | 0.06 | [0.88, 1.11] |
| Total effect of X on Y | 0.64 | 0.06 | [0.53, 0.75] | 0.71 | 0.06 | [0.59, 0.83] | 0.53 | 0.05 | [0.43, 0.64] |
| Indirect effect of X on Y via M_1_ | 0.05 | 0.02 | [0.01, 0.10] | 0.07 | 0.03 | [0.02, 0.12] | 0.01 | 0.02 | [-0.03, 0.06] |
| Indirect effect of X on Y via M_2_ | 0.31 | 0.04 | [0.23, 0.39] | 0.35 | 0.05 | [0.26, 0.44] | 0.25 | 0.04 | [0.19, 0.32] |
| Indirect effect of X on Y via M_1_ and M_2_ | 0.16 | 0.02 | [0.12, 0.21] | 0.19 | 0.02 | [0.14, 0.24] | 0.14 | 0.02 | [0.10, 0.17] |

*Note.* All reported effects were based on 5000 bootstrapping samples.

**Table S4**
*Results from Serial Mediation Analyses via Ought Self-Discrepancy and Positive Affect in Study 1*

| Pathway | *b* | *SE* | *95% CI* |  |  |  |  |  |  |
| --- | --- | --- | --- | --- | --- | --- | --- | --- | --- |
| *X* (PGFW); *M_1_* (Ought self-discrepancy); *M_2_* (Positive affect) | | | | | | | | | |
| Effect of X on M_1_ (Path a_1_) | -0.40 | 0.03 | [-0.46, -0.33] |  |  |  |  |  |  |
| Effect of X on M_2_ (Path a_2_) | 0.26 | 0.03 | [0.19, 0.32] |  |  |  |  |  |  |
| Effect of M_1_ on M_2_ (Path d_21_) | -0.34 | 0.03 | [-0.40, -0.28] |  |  |  |  |  |  |
|  | *Y* (Exhaustion) | | | *Y* (Cynicism) | | | *Y* (Professional efficacy) | | |
|  | *b* | *SE* | *95% CI* | *b* | *SE* | *95% CI* | *b* | *SE* | *95% CI* |
| Direct Effect of X on Y (Path c’) | -0.10 | 0.07 | [-0.23, 0.04] | -0.09 | 0.06 | [-0.21, 0.04] | 0.02 | 0.05 | [-0.07, 0.12] |
| Direct Effect of M_1_ on Y (Path b_1_) | 0.10 | 0.07 | [-0.04, 0.24] | -0.04 | 0.06 | [-0.16, 0.09] | -0.06 | 0.05 | [-0.15, 0.03] |
| Direct Effect of M_2_ on Y (Path b_2_) | -0.54 | 0.08 | [-0.70, -0.38] | -0.59 | 0.06 | [-0.71, -0.46] | 0.66 | 0.05 | [0.56, 0.75] |
| Total effect of X on Y | -0.35 | 0.06 | [-0.47, -0.24] | -0.31 | 0.05 | [-0.42, -0.20] | 0.29 | 0.04 | [0.21, 0.38] |
| Indirect effect of X on Y via M_1_ | -0.04 | 0.03 | [-0.10, 0.01] | 0.01 | 0.02 | [-0.02, 0.04] | 0.02 | 0.01 | [-0.01, 0.04] |
| Indirect effect of X on Y via M_2_ | -0.14 | 0.03 | [-0.19, -0.09] | -0.18 | 0.03 | [-0.24, -0.13] | 0.21 | 0.03 | [0.16, 0.26] |
| Indirect effect of X on Y via M_1_ and M_2_ | -0.07 | 0.01 | [-0.10, -0.05] | -0.04 | 0.01 | [-0.07, -0.03] | 0.05 | 0.01 | [0.03, 0.07] |

*Continued*

|  | *Y* (Vigor) | | | *Y* (Dedication) | | | *Y* (Absorption) | | |
| --- | --- | --- | --- | --- | --- | --- | --- | --- | --- |
|  | *b* | *SE* | *95% CI* | *b* | *SE* | *95% CI* | *b* | *SE* | *95% CI* |
| Direct Effect of X on Y (Path c’) | 0.13 | 0.05 | [0.03, 0.24] | 0.14 | 0.06 | [0.03, 0.25] | 0.13 | 0.06 | [0.02, 0.24] |
| Direct Effect of M_1_ on Y (Path b_1_) | -0.11 | 0.05 | [-0.22, -0.01] | -0.05 | 0.06 | [-0.17, 0.06] | -0.01 | 0.06 | [-0.13, 0.10] |
| Direct Effect of M_2_ on Y (Path b_2_) | 1.22 | 0.05 | [1.12, 1.32] | 1.42 | 0.05 | [1.31, 1.52] | 1.01 | 0.05 | [0.90, 1.12] |
| Total effect of X on Y | 0.64 | 0.06 | [0.53, 0.75] | 0.71 | 0.06 | [0.59, 0.83] | 0.53 | 0.05 | [0.42, 0.64] |
| Indirect effect of X on Y via M_1_ | 0.03 | 0.01 | [0.002, 0.06] | 0.01 | 0.02 | [-0.02, 0.05] | 0.004 | 0.02 | [-0.03, 0.04] |
| Indirect effect of X on Y via M_2_ | 0.38 | 0.04 | [0.30, 0.47] | 0.45 | 0.05 | [0.35, 0.54] | 0.32 | 0.04 | [0.25, 0.39] |
| Indirect effect of X on Y via M_1_ and M_2_ | 0.09 | 0.02 | [0.06, 0.13] | 0.11 | 0.02 | [0.07, 0.15] | 0.08 | 0.01 | [0.05, 0.11] |

*Note.* All reported effects were based on 5000 bootstrapping samples.
**Table S5**
*Results from Serial Mediation Analyses via Ideal Self-Discrepancy and Negative Affect in Study 2*

| Pathway | *b* | *SE* | *95% CI* |  |  |  |  |  |  |
| --- | --- | --- | --- | --- | --- | --- | --- | --- | --- |
| *X* (PGFW); *M_1_* (Ideal self-discrepancy); *M_2_* (Negative affect) | | | | | | | | | |
| Effect of X on M_1_ (Path a_1_) | -0.34 | 0.10 | [-0.52, -0.14] |  |  |  |  |  |  |
| Effect of X on M_2_ (Path a_2_) | -0.11 | 0.06 | [-0.22, 0.01] |  |  |  |  |  |  |
| Effect of M_1_ on M_2_ (Path d_21_ ) | 0.14 | 0.04 | [0.05, 0.22] |  |  |  |  |  |  |
|  | *Y* (Emotional Exhaustion) | | | *Y* (Depersonalization) | | | *Y* (Personal accomplishment) | | |
|  | *b* | *SE* | *95% CI* | *b* | *SE* | *95% CI* | *b* | *SE* | *95% CI* |
| Direct Effect of X on Y (Path c’) | -0.25 | 0.08 | [-0.43, -0.11] | -0.23 | 0.10 | [-0.43, -0.04] | 0.21 | 0.06 | [0.09, 0.33] |
| Direct Effect of M_1_ on Y (Path b_1_) | 0.08 | 0.05 | [-0.03, 0.17] | 0.06 | 0.06 | [-0.05, 0.17] | -0.15 | 0.04 | [-0.22, -0.07] |
| Direct Effect of M_2_ on Y (Path b_2_) | 0.82 | 0.09 | [0.63, 1.00] | 0.38 | 0.10 | [0.19, 0.57] | -0.07 | 0.07 | [-0.20, 0.06] |
| Total effect of X on Y | -0.40 | 0.09 | [-0.58, -0.23] | -0.31 | 0.08 | [-0.47, -0.15] | 0.27 | 0.06 | [0.16, 0.38] |
| Indirect effect of X on Y via M_1_ | -0.03 | 0.02 | [-0.07, 0.01] | -0.02 | 0.02 | [-0.07, 0.02] | 0.05 | 0.02 | [0.02, 0.09] |
| Indirect effect of X on Y via M_2_ | -0.09 | 0.05 | [-0.18, 0.01] | -0.04 | 0.02 | [-0.09, 0.004] | 0.01 | 0.01 | [-0.01, 0.03] |
| Indirect effect of X on Y via M_1_ and M_2_ | -0.04 | 0.02 | [-0.08, -0.01] | -0.02 | 0.01 | [-0.04, -0.003] | 0.003 | 0.004 | [-0.003, 0.01] |

*Continued*

|  | *Y* (Vigor) | | | *Y* (Dedication) | | | *Y* (Absorption) | | |
| --- | --- | --- | --- | --- | --- | --- | --- | --- | --- |
|  | *b* | *SE* | *95% CI* | *b* | *SE* | *95% CI* | *b* | *SE* | *95% CI* |
| Direct Effect of X on Y (Path c’) | 0.47 | 0.09 | [0.30, 0.65] | 0.37 | 0.10 | [0.19, 0.56] | 0.36 | 0.09 | [0.20, 0.54] |
| Direct Effect of M_1_ on Y (Path b_1_) | -0.26 | 0.06 | [-0.38, -0.14] | -0.28 | 0.06 | [-0.40, -0.16] | -0.27 | 0.05 | [-0.37, -0.17] |
| Direct Effect of M_2_ on Y (Path b_2_) | -0.28 | 0.10 | [-0.47, -0.07] | 0.04 | 0.10 | [-0.16, 0.23] | 0.07 | 0.10 | [-0.13, 0.26] |
| Total effect of X on Y | 0.60 | 0.09 | [0.42, 0.78] | 0.46 | 0.09 | [0.28, 0.64] | 0.44 | 0.08 | [0.28, 0.61] |
| Indirect effect of X on Y via M_1_ | 0.09 | 0.03 | [0.03, 0.16] | 0.10 | 0.04 | [0.03, 0.17] | 0.09 | 0.03 | [0.03, 0.15] |
| Indirect effect of X on Y via M_2_ | 0.03 | 0.02 | [-0.004, 0.08] | -0.004 | 0.01 | [-0.04, 0.02] | -0.01 | 0.01 | [-0.04, 0.01] |
| Indirect effect of X on Y via M_1_ and M_2_ | 0.01 | 0.01 | [0.001, 0.03] | -0.002 | 0.01 | [-0.01, 0.01] | -0.003 | 0.01 | [-0.02, 0.01] |

*Note.* Path a_1,_ Path a_2_ and Path d_21_ vary slightly across the three engagement dimensions due to missing data. All reported effects were based on 5000 bootstrapping samples.

**Table S6**
*Results from Serial Mediation Analyses via Ought Self-Discrepancy and Negative Affect in Study 2*

| Pathway | *b* | *SE* | *95% CI* |  |  |  |  |  |  |
| --- | --- | --- | --- | --- | --- | --- | --- | --- | --- |
| *X* (PGFW); *M_1_* (Ought self-discrepancy); *M_2_* (Negative affect) | | | | | | | | | |
| Effect of X on M_1_ (Path a_1_) | -0.29 | 0.11 | [-0.50, -0.07] |  |  |  |  |  |  |
| Effect of X on M_2_ (Path a_2_) | -0.12 | 0.06 | [-0.24, -0.01] |  |  |  |  |  |  |
| Effect of M_1_ on M_2_ (Path d_21_ ) | 0.10 | 0.03 | [0.04, 0.16] |  |  |  |  |  |  |
|  | *Y* (Emotional Exhaustion) | | | *Y* (Depersonalization) | | | *Y* (Personal accomplishment) | | |
|  | *b* | *SE* | *95% CI* | *b* | *SE* | *95% CI* | *b* | *SE* | *95% CI* |
| Direct Effect of X on Y (Path c’) | -0.26 | 0.08 | [-0.43, -0.11] | -0.23 | 0.10 | [-0.42, -0.05] | 0.23 | 0.06 | [0.12, 0.35] |
| Direct Effect of M_1_ on Y (Path b_1_) | 0.06 | 0.05 | [-0.03, 0.15] | 0.08 | 0.04 | [-0.0004, 0.16] | -0.08 | 0.03 | [-0.14, -0.01] |
| Direct Effect of M_2_ on Y (Path b_2_) | 0.82 | 0.10 | [0.62, 1.01] | 0.36 | 0.09 | [0.19, 0.54] | -0.09 | 0.07 | [-0.23, 0.03] |
| Total effect of X on Y | -0.40 | 0.09 | [-0.58, -0.23] | -0.31 | 0.08 | [-0.47, -0.15] | 0.27 | 0.06 | [0.16, 0.38] |
| Indirect effect of X on Y via M_1_ | -0.02 | 0.02 | [-0.06, 0.01] | -0.02 | 0.02 | [-0.06, 0.0004] | 0.02 | 0.01 | [0.002, 0.05] |
| Indirect effect of X on Y via M_2_ | -0.10 | 0.05 | [-0.20, -0.004] | -0.05 | 0.02 | [-0.09, -0.002] | 0.01 | 0.01 | [-0.005, 0.03] |
| Indirect effect of X on Y via M_1_ and M_2_ | -0.02 | 0.01 | [-0.05, -0.004] | -0.01 | 0.01 | [-0.02, -0.002] | 0.003 | 0.003 | [-0.001, 0.01] |

*Continued*

|  | *Y* (Vigor) | | | *Y* (Dedication) | | | *Y* (Absorption) | | |
| --- | --- | --- | --- | --- | --- | --- | --- | --- | --- |
|  | *b* | *SE* | *95% CI* | *b* | *SE* | *95% CI* | *b* | *SE* | *95% CI* |
| Direct Effect of X on Y (Path c’) | 0.52 | 0.10 | [0.33, 0.71] | 0.42 | 0.10 | [0.24, 0.61] | 0.42 | 0.09 | [0.25, 0.60] |
| Direct Effect of M_1_ on Y (Path b_1_) | -0.10 | 0.05 | [-0.20, 0.01] | -0.11 | 0.06 | [-0.22, -0.002] | -0.07 | 0.04 | [-0.16, 0.01] |
| Direct Effect of M_2_ on Y (Path b_2_) | -0.34 | 0.11 | [-0.54, -0.12] | -0.03 | 0.11 | [-0.23, 0.19] | -0.01 | 0.10 | [-0.22, 0.19] |
| Total effect of X on Y | 0.60 | 0.09 | [0.42, 0.78] | 0.46 | 0.09 | [0.28, 0.64] | 0.44 | 0.08 | [0.28, 0.61] |
| Indirect effect of X on Y via M_1_ | 0.03 | 0.02 | [-0.002, 0.07] | 0.03 | 0.02 | [-0.0001, 0.08] | 0.02 | 0.02 | [-0.004, 0.06] |
| Indirect effect of X on Y via M_2_ | 0.04 | 0.03 | [0.001, 0.10] | 0.00 | 0.01 | [-0.03, 0.03] | 0.001 | 0.01 | [-0.03, 0.03] |
| Indirect effect of X on Y via M_1_ and M_2_ | 0.01 | 0.01 | [0.001, 0.02] | 0.001 | 0.003 | [-0.01, 0.01] | 0.0003 | 0.003 | [-0.01, 0.01] |

Note. Path a1, Path a2 and Path d21 vary slightly across the three engagement dimensions due to missing data. All reported effects were based on 5000 bootstrapping samples.

**Table S7**
*Results from Serial Mediation Analyses via Ideal Self-Discrepancy and Positive Affect in Study 2*

| Pathway | *b* | *SE* | *95% CI* |  |  |  |  |  |  |
| --- | --- | --- | --- | --- | --- | --- | --- | --- | --- |
| *X* (PGFW); *M_1_* (Ideal self-discrepancy); *M_2_* (Positive affect) | | | | | | | | | |
| Effect of X on M_1_ (Path a_1_) | -0.34 | 0.10 | [-0.52, -0.14] |  |  |  |  |  |  |
| Effect of X on M_2_ (Path a_2_) | 0.23 | 0.05 | [0.14, 0.33] |  |  |  |  |  |  |
| Effect of M_1_ on M_2_ (Path d_21_ ) | -0.16 | 0.03 | [-0.22, -0.09] |  |  |  |  |  |  |
|  | *Y* (Emotional Exhaustion) | | | *Y* (Depersonalization) | | | *Y* (Personal accomplishment) | | |
|  | *b* | *SE* | *95% CI* | *b* | *SE* | *95% CI* | *b* | *SE* | *95% CI* |
| Direct Effect of X on Y (Path c’) | -0.20 | 0.09 | [-0.38, 0.02] | -0.19 | 0.10 | [-0.40, 0.02] | 0.09 | 0.06 | [-0.02, 0.20] |
| Direct Effect of M_1_ on Y (Path b_1_) | 0.09 | 0.06 | [-0.02, 0.20] | 0.05 | 0.05 | [-0.05, 0.16] | -0.07 | 0.04 | [-0.14, -0.004] |
| Direct Effect of M_2_ on Y (Path b_2_) | -0.61 | 0.11 | [-0.83, -0.38] | -0.37 | 0.11 | [-0.57, -0.14] | 0.53 | 0.07 | [0.40, 0.66] |
| Total effect of X on Y | -0.40 | 0.09 | [-0.58, -0.23] | -0.31 | 0.08 | [-0.47, -0.15] | 0.27 | 0.06 | [0.16, 0.38] |
| Indirect effect of X on Y via M_1_ | -0.03 | 0.02 | [-0.08, 0.01] | -0.02 | 0.02 | [-0.06, 0.02] | 0.03 | 0.01 | [0.001, 0.06] |
| Indirect effect of X on Y via M_2_ | -0.14 | 0.04 | [-0.23, -0.07] | -0.09 | 0.03 | [-0.16, -0.03] | 0.12 | 0.03 | [0.07, 0.19] |
| Indirect effect of X on Y via M_1_ and M_2_ | -0.03 | 0.01 | [-0.06, -0.01] | -0.02 | 0.01 | [-0.04, -0.005] | 0.03 | 0.01 | [0.01, 0.05] |

*Continued*

|  | *Y* (Vigor) | | | *Y* (Dedication) | | | *Y* (Absorption) | | |
| --- | --- | --- | --- | --- | --- | --- | --- | --- | --- |
|  | *b* | *SE* | *95% CI* | *b* | *SE* | *95% CI* | *b* | *SE* | *95% CI* |
| Direct Effect of X on Y (Path c’) | 0.26 | 0.08 | [0.09, 0.43] | 0.08 | 0.07 | [-0.06, 0.23] | 0.19 | 0.08 | [0.03, 0.35] |
| Direct Effect of M_1_ on Y (Path b_1_) | -0.14 | 0.05 | [-0.23, -0.04] | -0.08 | 0.04 | [-0.17, -0.004] | -0.15 | 0.05 | [-0.25, -0.05] |
| Direct Effect of M_2_ on Y (Path b_2_) | 1.06 | 0.10 | [0.88, 1.25] | 1.21 | 0.09 | [1.03, 1.39] | 0.73 | 0.10 | [0.54, 0.93] |
| Total effect of X on Y | 0.60 | 0.09 | [0.42, 0.78] | 0.46 | 0.09 | [0.28, 0.64] | 0.44 | 0.08 | [0.28, 0.61] |
| Indirect effect of X on Y via M_1_ | 0.04 | 0.02 | [0.01, 0.10] | 0.03 | 0.02 | [0.001, 0.07] | 0.05 | 0.02 | [0.01, 0.10] |
| Indirect effect of X on Y via M_2_ | 0.24 | 0.06 | [0.14, 0.36] | 0.28 | 0.07 | [0.16, 0.42] | 0.17 | 0.04 | [0.09, 0.26] |
| Indirect effect of X on Y via M_1_ and M_2_ | 0.05 | 0.02 | [0.02, 0.10] | 0.06 | 0.02 | [0.02, 0.11] | 0.04 | 0.01 | [0.01, 0.07] |

*Note.* Path a_1,_ Path a_2_ and Path d_21_ vary slightly across the three engagement dimensions due to missing data. All reported effects were based on 5000 bootstrapping samples.

**Table S8**

*Results from Serial Mediation Analyses via Ought/Actual Self-Discrepancy and Positive Affect for Burnout Dimensions in Study 2*

| Pathway | *b* | *SE* | *95% CI* |  |  |  |  |  |  |
| --- | --- | --- | --- | --- | --- | --- | --- | --- | --- |
| *X* (PGFW); *M_1_* (Ought self-discrepancy); *M_2_* (Positive affect) | | | | | | | | | |
| Effect of X on M_1_ (Path a_1_) | -0.29 | 0.11 | [-0.5, -0.07] |  |  |  |  |  |  |
| Effect of X on M_2_ (Path a_2_) | 0.27 | 0.05 | [0.18, 0.37] |  |  |  |  |  |  |
| Effect of M_1_ on M_2_ (Path d_21_ ) | -0.05 | 0.03 | [-0.11, 0.01] |  |  |  |  |  |  |
|  | *Y* (Emotional Exhaustion) | | | *Y* (Depersonalization) | | | *Y* (Personal accomplishment) | | |
|  | *b* | *SE* | *95% CI* | *b* | *SE* | *95% CI* | *b* | *SE* | *95% CI* |
| Direct Effect of X on Y (Path c’) | -0.19 | 0.09 | [-0.37, -0.02] | -0.18 | 0.10 | [-0.38, 0.02] | 0.09 | 0.06 | [-0.02, 0.21] |
| Direct Effect of M_1_ on Y (Path b_1_) | 0.11 | 0.05 | [0.02, 0.21] | 0.10 | 0.04 | [0.02, 0.18] | -0.06 | 0.03 | [-0.11, -0.004] |
| Direct Effect of M_2_ on Y (Path b_2_) | -0.63 | 0.11 | [-0.85, -0.40] | -0.37 | 0.11 | [-0.57, -0.15] | 0.55 | 0.06 | [0.43, 0.68] |
| Total effect of X on Y | -0.40 | 0.09 | [-0.58, -0.23] | -0.31 | 0.08 | [-0.47, -0.15] | 0.27 | 0.06 | [0.16, 0.38] |
| Indirect effect of X on Y via M_1_ | -0.03 | 0.02 | [-0.08, -0.003] | -0.03 | 0.02 | [-0.07, -0.002] | 0.02 | 0.01 | [0.0005, 0.04] |
| Indirect effect of X on Y via M_2_ | -0.17 | 0.05 | [-0.27, -0.09] | -0.10 | 0.04 | [-0.18, -0.04] | 0.15 | 0.03 | [0.09, 0.22] |
| Indirect effect of X on Y via M_1_ and M_2_ | -0.01 | 0.01 | [-0.03, 0.001] | -0.01 | 0.005 | [-0.02, 0.001] | 0.01 | 0.01 | [-0.001, 0.02] |

*Continued*

|  | *Y* (Vigour) | | | *Y* (Dedication) | | | *Y* (Absorption) | | |
| --- | --- | --- | --- | --- | --- | --- | --- | --- | --- |
|  | *b* | *SE* | *95% CI* | *b* | *SE* | *95% CI* | *b* | *SE* | *95% CI* |
| Direct Effect of X on Y (Path c’) | 0.27 | 0.09 | [0.10, 0.44] | 0.08 | 0.07 | [-0.06, 0.23] | 0.21 | 0.08 | [0.05, 0.37] |
| Direct Effect of M_1_ on Y (Path b_1_) | -0.07 | 0.04 | [-0.15, 0.0003] | -0.05 | 0.04 | [-0.12, 0.02] | -0.03 | 0.04 | [-0.10, 0.04] |
| Direct Effect of M_2_ on Y (Path b_2_) | 1.12 | 0.09 | [0.94, 1.30] | 1.24 | 0.08 | [1.07, 1.41] | 0.81 | 0.10 | [0.62, 0.99] |
| Total effect of X on Y | 0.60 | 0.09 | [0.42, 0.78] | 0.46 | 0.09 | [0.28, 0.64] | 0.44 | 0.08 | [0.28, 0.61] |
| Indirect effect of X on Y via M_1_ | 0.02 | 0.01 | [-0.001, 0.05] | 0.01 | 0.01 | [-0.01, 0.04] | 0.01 | 0.01 | [-0.01, 0.03] |
| Indirect effect of X on Y via M_2_ | 0.29 | 0.06 | [0.18, 0.42] | 0.34 | 0.07 | [0.21, 0.48] | 0.21 | 0.05 | [0.12, 0.32] |
| Indirect effect of X on Y via M_1_ and M_2_ | 0.02 | 0.01 | [-0.002, 0.05] | 0.02 | 0.01 | [-0.002, 0.05] | 0.01 | 0.01 | [-0.001, 0.03] |

*Note.* Path a_1,_ Path a_2_ and Path d_21_ vary slightly across the three engagement dimensions due to missing data.

Table S9. Effect of Self-Affirmation Manipulation on All Study 2 Variables.

|  | Variables | Self-Affirmation Condition  *M* (*SD*) | | Control  Condition  *M* (*SD*) | | *t*-value | | *p*-value | |
| --- | --- | --- | --- | --- | --- | --- | --- | --- | --- |
| 1. PGFW | | 2.92 (0.83) | 2.97 (0.81) | | -0.49 | | .62 | |  |
| 2. Emotional exhaustion | | 4.28 (1.17) | 4.34 (1.16) | | -0.39 | | .70 | |  |
| 3. Depersonalization | | 3.03 (1.11) | 3.02 (0.98) | | 0.056 | | .96 | |  |
| 4. Personal accomplishment | | 4.61 (0.74) | 4.66 (0.74) | | -0.55 | | .58 | |  |
| 5. Vigor | | 3.38 (1.18) | 3.46 (1.30) | | -0.45 | | .65 | |  |
| 6. Dedication | | 4.73 (1.21) | 4.72 (1.18) | | 0.098 | | .92 | |  |
| 7. Absorption | | 4.26 (1.07) | 4.35 (1.09) | | -0.63 | | .53 | |  |
| 8. Ideal   self-discrepancy | | 3.74 (1.33) | 3.57 (1.15) | | 1.03 | | .30 | |  |
| 9. Ought   self-discrepancy | | 3.67 (1.47) | 3.43 (1.48) | | 1.25 | | .21 | |  |
| 10. Positive affect | | 3.18 (0.70) | 3.17 (0.66) | | 0.19 | | .85 | |  |
| 11. Negative affect | | 2.17 (0.70) | 2.18 (0.73) | | -0.090 | | .93 | |  |
